# Integration of Swin UNETR and statistical shape modeling for a semi-automated segmentation of the knee and biomechanical modeling of articular cartilage

**DOI:** 10.1038/s41598-024-52548-9

**Published:** 2024-02-02

**Authors:** Reza Kakavand, Mehrdad Palizi, Peyman Tahghighi, Reza Ahmadi, Neha Gianchandani, Samer Adeeb, Roberto Souza, W. Brent Edwards, Amin Komeili

**Affiliations:** 1 Department of Biomedical Engineering, Schulich School of Engineering, University of Calgary, CCIT 216, 2500 University Drive NW, Calgary, AB T2N 1N4 Canada; 2 Civil and Environmental Engineering Department, Faculty of Engineering, University of Alberta, Edmonton, Canada; 3 Department of Electrical and Software Engineering, Schulich School of Engineering, University of Calgary, Calgary, Canada; 4 Cumming School of Medicine, Hotchkiss Brain Institute, University of Calgary, Calgary, Canada; 5 McCaig Institute for Bone and Joint Health, University of Calgary, Calgary, Canada; 6 Human Performance Laboratory, Faculty of Kinesiology, University of Calgary, Calgary, Canada

**Keywords:** Biomedical engineering, Computational science

## Abstract

Simulation studies, such as finite element (FE) modeling, provide insight into knee joint mechanics without patient involvement. Generic FE models mimic the biomechanical behavior of the tissue, but overlook variations in geometry, loading, and material properties of a population. Conversely, subject-specific models include these factors, resulting in enhanced predictive precision, but are laborious and time intensive. The present study aimed to enhance subject-specific knee joint FE modeling by incorporating a semi-automated segmentation algorithm using a 3D Swin UNETR for an initial segmentation of the femur and tibia, followed by a statistical shape model (SSM) adjustment to improve surface roughness and continuity. For comparison, a manual FE model was developed through manual segmentation (i.e., the de-facto standard approach). Both FE models were subjected to gait loading and the predicted mechanical response was compared. The semi-automated segmentation achieved a Dice similarity coefficient (DSC) of over 98% for both the femur and tibia. Hausdorff distance (mm) between the semi-automated and manual segmentation was 1.4 mm. The mechanical results (max principal stress and strain, fluid pressure, fibril strain, and contact area) showed no significant differences between the manual and semi-automated FE models, indicating the effectiveness of the proposed semi-automated segmentation in creating accurate knee joint FE models. We have made our semi-automated models publicly accessible to support and facilitate biomechanical modeling and medical image segmentation efforts (https://data.mendeley.com/datasets/k5hdc9cz7w/1).

## Introduction

Simulation studies, such as finite element (FE) modeling, provide insight into the stresses, strains, and contact mechanics within the knee joint under physiologically relevant loading conditions^1–7^. Generic FE models typically predict the biomechanical behavior of the knee joint based on representative or aggregate data^8^ from a cohort of subjects,allowing researchers to create a simplified and standardized representation of the biological system or mechanical structure under investigation as a fundamental starting point for research studies. Alternatively, subject-specific FE models include personalized information resulting in more accurate predictions^9–14^, but require labor- and time-intensive manual segmentation of computed tomography (CT) and magnetic resonance images (MRI)^15,16^, with limited reproducibility^17–19^.

Convolutional neural network (CNN) and statistical shape models (SSM) have demonstrated the ability to accelerate segmentation operations of medical images. SSM involves a principal component analysis (PCA), performed on a training set of extracted subject geometries to determine its modes of spatial variation^20–23^. Different CNNs and SSMs have succeeded at various segmentation tasks^24^. Ambellan et al.^25^ used CNN for 2D and 3D segmentation of knee tissues and implemented SSM to control regions with abnormal shapes. The Dice similarity coefficient (DSC) values for the femur and tibia were 98.6% and 98.5% using data from the Osteoarthritis Initiative (OAI) (https://nda.nih.gov/oai/). Paproki et al.^26^ and Tack et al.^27^ facilitated the segmentation of menisci in healthy and OA knees using active shape modeling and SSM. Deep Convolutional Neural Networks (DNNs), particularly the U-Net model, have demonstrated exceptional performance in medical image segmentation across different modalities and organs^28,29^. However, CNN-based approaches often struggle to capture long-range dependencies (the influence of pixels or regions that are spatially distant from each other) due to their reliance on localized receptive fields (the image segment for the convolution)^30^.

Recent machine learning methods have significantly improved the segmentation of organs from biomedical images. For instance, UNETR is a neural network architecture that combines the strengths of UNet and transformer models for accurate image segmentation tasks^30^. Swin UNETR^31^ was developed for segmenting brain tumors from MRI and demonstrated superior accuracy and efficiency in a variety of benchmarks. Swin UNETR combines the encoder from Swin transformers, a modified version of Vision Transformer (ViT), with a decoder inspired by 3D U-Net^32,33^. Swin transformers, specialized for the visual domain, overcame the quadratic model complexity drawback of ViT by employing a shifted windowing scheme. The hierarchical structure of Swin transformers allows for modeling and combining image features at multiple scales, similar to CNNs. Furthermore, the linear computational complexity of Swin transformers enhances their efficiency for dense prediction tasks using high-resolution images^34^. Nevertheless, the outcome of the automatic segmentation methods requires manual correction to improve surface smoothness, fill holes, and correct abnormal morphologies. Therefore, 3D FE model preparation from biomedical images still requires significant human intervention and supervision, with the potential to introduce bias. Taking advantage of recent advances in medical image segmentation, the previous algorithms for knee joint cartilage segmentation could be revisited to enhance their accuracy and reproducibility for computational modeling. A gap exists between the existing advanced automated segmentation models and their implementation in biomechanical modeling. In addition, there seems to be a lack of publicly available automated segmentation models suitable for subject-specific biomechanical modeling of knee joints.

This study aimed to develop an advanced semi-automated segmentation method for creating knee joint FE models. The objectives of this study were: (1) to train a 3D Swin UNETR transformer and SSM for the semi-automated segmentation of distal femur and proximal tibia, which is suitable for biomechanical modeling, and (2) to assess the FE model outcome, including maximum principal stress and strain, interstitial fluid pressure, fibril strain, and contact area, predicted by the semiautomated model and the manually segmented model. We have made our semi-automated models publicly accessible to support the community and facilitate biomechanical modeling and medical image segmentation efforts (https://data.mendeley.com/datasets/k5hdc9cz7w/1).

## Method

**Figure 1:**
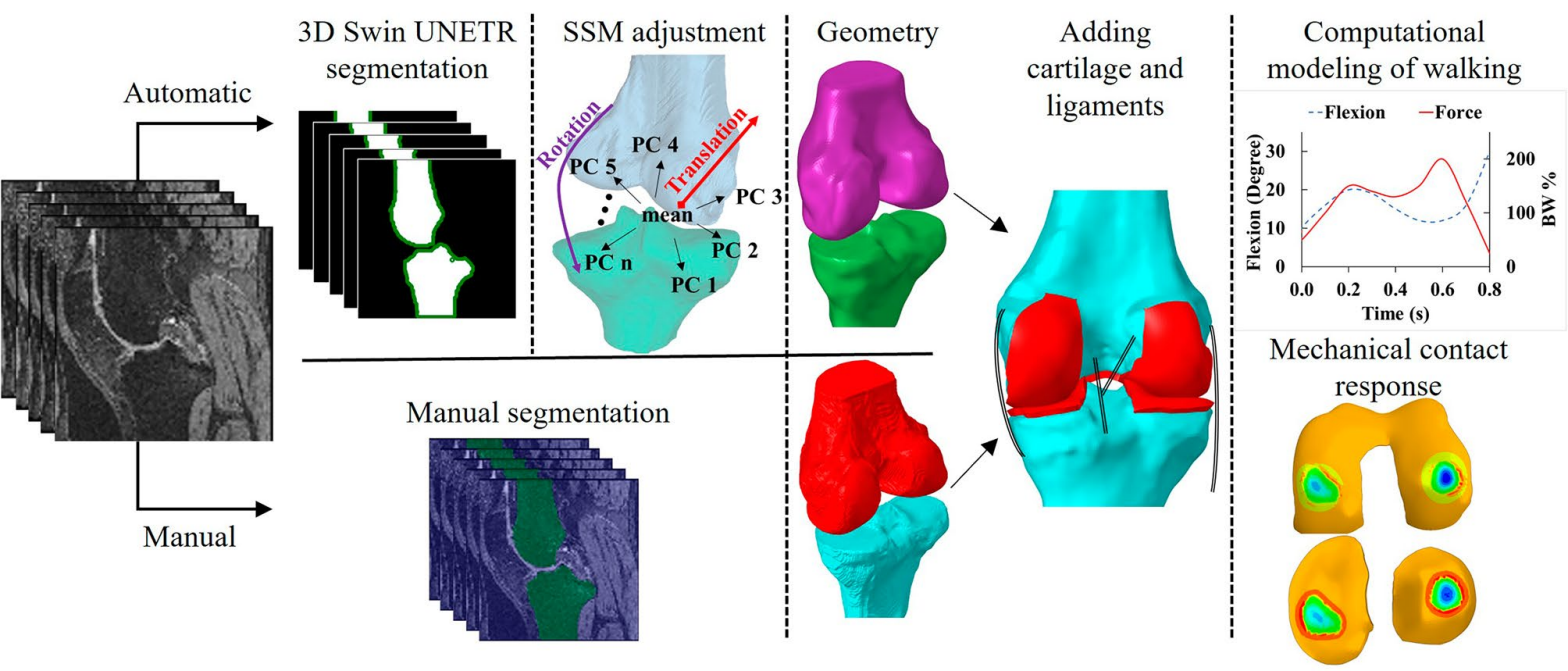
FE model development workflow used in this study. Semi-automated and manual segmentations were developed from MRIs of knee joints from OAI database (https://nda.nih.gov/oai/). A SSM model of knee joint was registered to the geometry outcome of 3D Swin UNETR, giving smooth surfaces suitable for mesh generation for FE analysis. A gait loading was applied to both manual and semi-automated models, and their mechanical responses were compared, including cartilage contact mechanics and pore pressure. PC: principal components.

Two FE models, manual and semi-automated, were generated and then applied to a total of nine knee MRIs, details of which were given in Section "Computational modeling". The geometry of the femur and tibia was the only difference between the manual and semi-automated FE models, created using either manual or semi-automated segmentation, respectively. The geometries for cartilages were manually segmented and added to both FE models. For the semi-automated segmentation, a 3D Swin UNETR transformer was used for the initial segmentation of femur and tibia, which was further adjusted with SSM to improve their surface quality in terms of surface roughness and hole filling. Bone surfaces (from both the manual and semi-automated FE models) were meshed using quadrilateral elements. The quadrilateral elements in the calcified region were extruded to the articular surface of the cartilage using hexahedral meshes. These hexahedral meshes represented cartilage in the FE models. Ligaments were modelled as bi-linear springs that could withstand tension but not compression (Fig. 1). The predicted mechanical response of the manual and semi-automated FE models, including the cartilage contact mechanics and pore pressure, were compared. Specific details of these procedures are outlined below.

### Data

MRIs from 507 individuals (61.9 ± 9.3 years; 29.27 ± 4.52 BMI [kg/m^2^]; 0.36 × 0.36 × 0.7 mm image resolution, 262 males and 245 females) were extracted from the Osteoarthritis Initiative (OAI) database (https://nda.nih.gov/oai/). Regions-of-interest (ROIs) for the femur, tibia and cartilage were segmented by skilled users from the Zuse Institute Berlin^25^. All grades of OA were included, but with a high tendency towards severe cases. Specifically, the dataset included 60 MRIs with Kellgren-Lawrence (KL) grade 0, 77 grade 1, 61 grade 2, 151 grade 3, and 158 MRIs with grade 4 OA^25^. To evaluate the performance of Swin UNETR and SSM models, we used randomly selected fivefold cross-validation (Fig. 1S in the supplementary material). Each fold had 405 MRIs for training and 102 MRIs for testing. To evaluate the performance of FE models, we used 9 randomly selected samples from the test set since FE modeling of 102 samples is extremely time-consuming and currently infeasible (until the automation of the steps in FE modeling of knee joint such as meshing, material property assigning and loading is achieved).

### Swin UNETR

**Figure 2:**
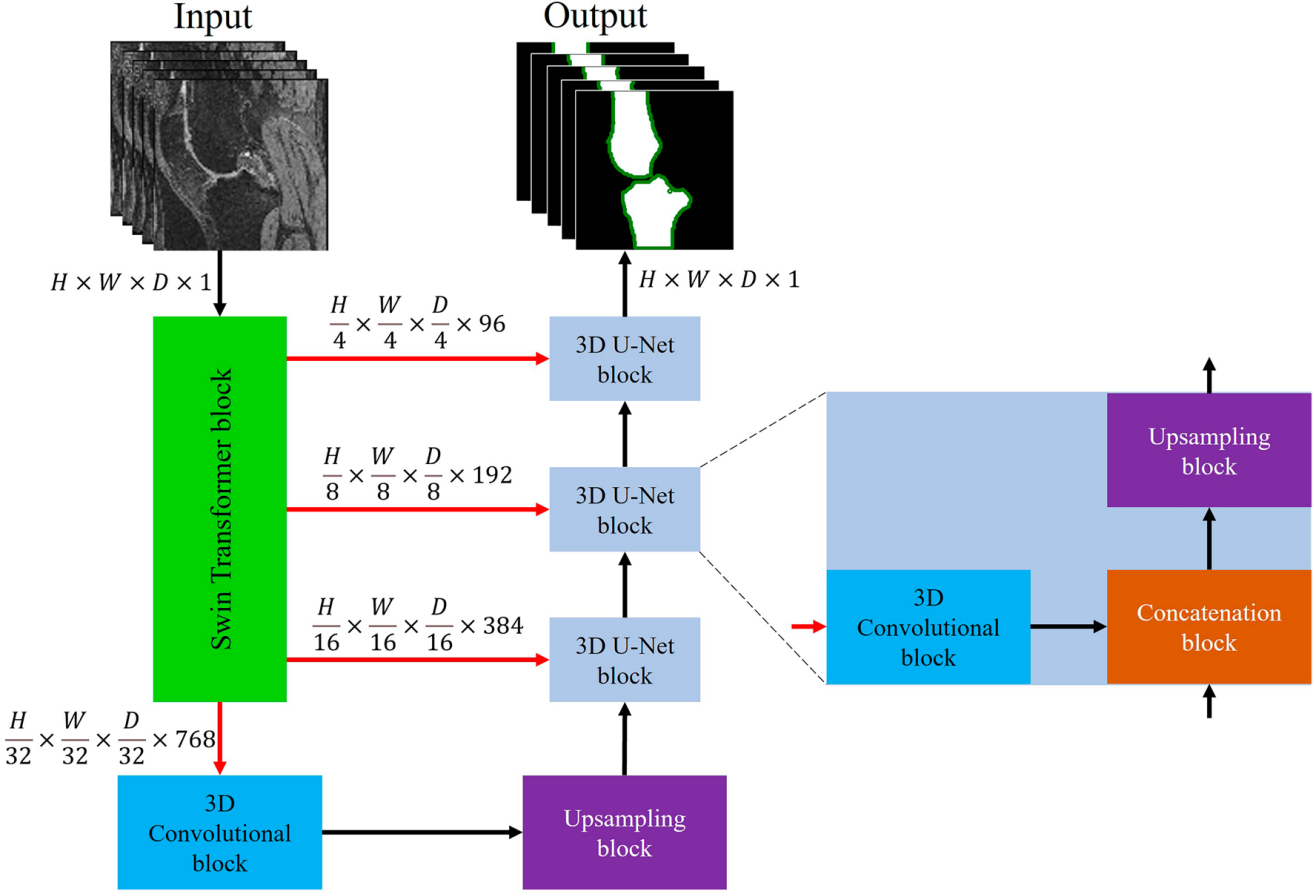
The structure of the employed Swin UNETR segmentation model. It was a combination of a Swin transformer as the encoder and 3D U-Net blocks as the decoder. Swin transformer extracted hierarchical representation from given MRIs and 3D U-Net utilized these representations to construct the segmentation mask. The model outputs a binary label for each pixel. Please note that for each femur and tibia, we developed a separate Swin UNETR segmentation model. Here, for the illustration, the outputs of femur and tibia were combined.

The hierarchical structure of the Swin transformer allows for modeling and combining image features at multiple scales (like CNNs), and it maintains linear computational complexity in relation to image size^34^. The four output features extracted from the Swin transformer (indicated by red arrows) were fed into the 3D U-Net blocks to reconstruct an image with the same size as the input (Fig. 2). The model yields a single output for each pixel. For 3D Swin UNETR, a patch size of 2, a window size of 7, and an initial feature size of 48 were used. The Swin transformer had four stages and utilized three 3D U-Net blocks for upsampling (Fig. 2). The Swin UNETR was trained using the DSC and binary cross entropy focal loss.

Our proposed method was implemented in Python, and our deep learning models were implemented using the Pytroch library. All models were trained with a batch size of 8, using the Adam optimizer^35,36^ with an initial learning rate of $0.0001$ and early stopping to avoid overfitting. The model was evaluated using fivefold cross-validation (Fig. 1S in the supplementary material). The original image size was 160,384,384 pixels. During training, each MRI was resized to $128 \times 128 \times 160$ pixels (to reduce the computational costs) and cropped to $96 \times 96 \times 96$ pixels from random regions. The resizing and cropping significantly improved the generalization of the Swin UNETR model. We applied window center adjustment on MRIs as the preprocessing step to translate them to the range [0,1]. The data augmentation and Swin UNETR implementation were done using the Monai library (https://docs.monai.io/en/stable/). All the other variables were the default settings in the Monai. The Swin UNETR models and codes have been made publicly available, so researchers can use these models or customize the code for a different dataset to meet their needs (https://data.mendeley.com/datasets/k5hdc9cz7w/1). The output of Swin UNETR, which is an image, needs to be manually converted to a CAD format (for example, using ITK-SNAP software), making this approach a semi-automated method. A tutorial video was recorded about converting segmented images to a CAD, which is available at https://data.mendeley.com/datasets/k5hdc9cz7w/1.

### SSM

Using SSM, a shape may be defined as:

1 $${\text{Shape}}={\text{M}}+{\text{PC}}\times {\text{b}}$$

where M is the mean of the points of the shape, PC (PC_1_, PC_2_, PC_3_, …) is the principal components (the modes of variations of the points of the shape), and b is a vector of weights. To build an SSM for the femur and tibia, correspondence was first established between the samples, and then, Principal Component Analysis (PCA) was employed to model anatomical variation. The SSM was developed with a custom Python script (available in our data at https://data.mendeley.com/datasets/k5hdc9cz7w/1). Constructing correspondence between the samples included coarse and fine alignment steps using point-set representation. First, a manually segmented mesh belonging to one of the subjects was selected as the initial template. The template mesh for the femur and tibia was refined into meshes with optimized tessellation quality using the iso-parametrization method^37^. The mesh was then smoothed using the Taubin method^38^. A dense set of points with uniform distribution was sampled on each mesh (representing the femur or tibia for a subject or the template). To uniformly sample points on the meshes, the Poisson-disk point-set sampling method^39^ was used to achieve well-distributed points, then the uniformization technique^40^ was used to further homogenize the distance between the neighboring points.

For each subject, the combined point set for the femur and tibia was coarsely aligned to the template using the centroid, the centroid size, and the principal axes of the combined point set^40^. Next, the template point set was matched on each sample. The matching process involved the rigid registration of the template to the sample, followed by a non-rigid registration. The coherent Point Drift (CPD) method^41^ was used for the rigid and non-rigid registration tasks. After registering the template to all samples, the redundant rigid transformation within the samples was removed using the Generalized Procrustes Analysis (GPA)^42^. The average shape for the femur and tibia was computed as the arithmetic mean of the point sets in correspondence (after applying GPA). To generate a mesh for each instance of the SSM, the deformation field between the average point set and the point set of the shape instance was decomposed into affine and non-rigid components using the Thin-Plate Spline (TPS) formulation^43^, and the characterized transformation was applied to the vertices of the average (with high quality of tessellation). This process resulted in a representation of each sample (femur or tibia) with a deformed version of the average mesh with high-quality tessellation.

### Cartilage extrusion

**Figure 3:**
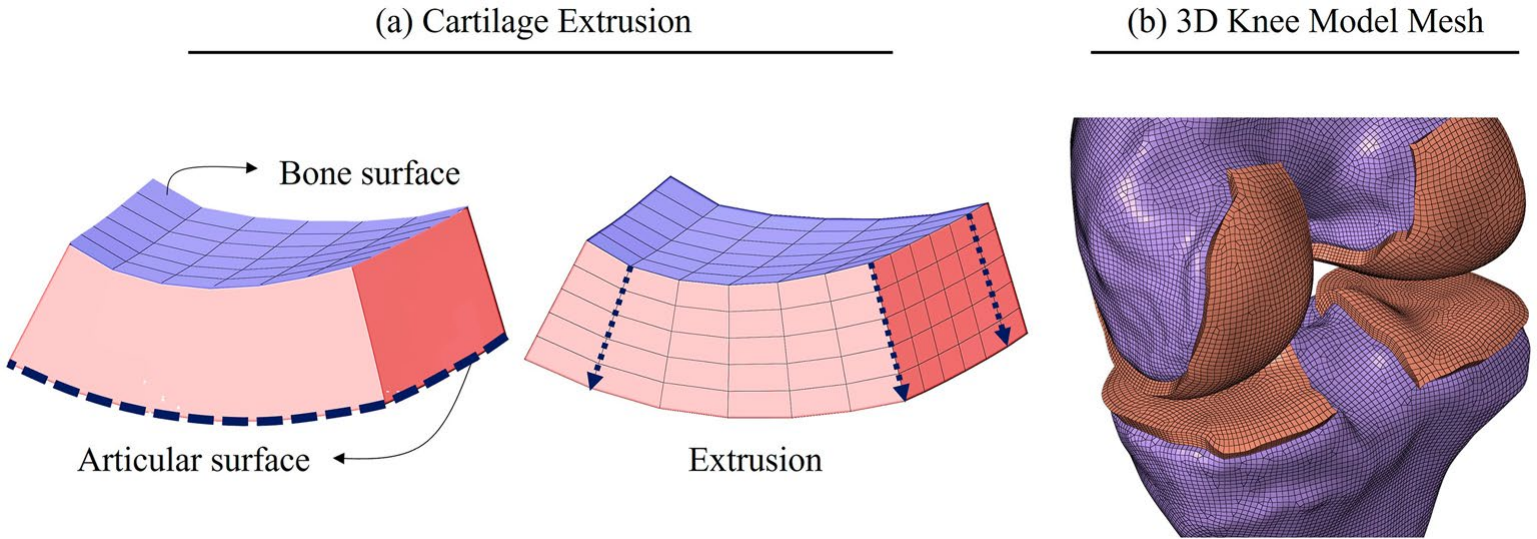
(**a**) Mapping the quadrilateral surface elements of the bone to the articular cartilage surface using solid elements. (**b**) 3D knee joint model.

The segmented femora and tibiae were meshed using quadrilateral elements. The femoral condyle and tibial plateau surfaces that share the calcified cartilage zone were mapped to the articular cartilage surface using 8 node solid elements, creating 5 layers of hexahedral elements (Fig. 3). In this way, the variation of cartilage thickness over the joint was captured, and common nodes were defined at the interface of bone and cartilage (a detailed explanation of meshing cartilage can be found in Fig. 2S in the supplementary material). This aimed to accelerate computational time and improve the convergence rate.

Mesh sensitivity was performed with three different element sizes of 2, 1, and 0.5 mm (coarse, fine, and very fine). A 1% change in the contact area and average contact pressure between models were used as the convergence criterion to select the optimized mesh size. The difference in outputs between fine and very fine elements was less than 1%, indicating successful convergence at the fine resolutions, which was selected for further FE analyses.

### Computational modeling

#### Material and finite element modeling

We employed the biphasic constitutive model proposed by Federico and Gasser^44^ and Federico and Grillo^45^ for cartilage, which consisted of an incompressible fluid phase and a fibril-reinforced solid/matrix phase. Collagen fibrils were separated into isotropic and anisotropic components under the assumption that the matrix was isotropic and inhomogeneous along its thickness. The directional orientation of the fibrillar network was captured by the anisotropic fibrils. Table 1S in the supplementary material provides a description of the biphasic model and associated material constants. The results of creep indentation experiments conducted by Pajerski^46^ and Athanasiou et al.^47^ on human knee cartilage served as the basis for the extracellular matrix (ECM) material properties^46^. A detailed explanation of the cartilage constitutive laws with its material constants used for describing cartilage behavior can be found in the supplementary material and also in our previous work^48,49^. Briefly, the state of stress was defined by:

2 $$\sigma =-pI+{\varnothing }_{0}{\sigma }_{0}+{\varnothing }_{1}({\sigma }_{1i}+{\sigma }_{1a})$$

where $\sigma$ is the total stress in the tissue, *p* is the hydrostatic interstitial fluid pressure, *I* is the unity tensor, and $\varnothing$ is the volume fraction. Here, subscripts 0 and 1 denote matrix and collagen fibrils, respectively. The matrix was considered isotropic, while the collagen fibrils were divided into isotropic (${\sigma }_{1i})$ and anisotropic (${\sigma }_{1a})$.

Hexahedral pore pressure elements (C3D8P) were used to define knee cartilage mesh. A surface-to-surface contact with frictionless tangential behavior was presented with the contact mechanics of cartilage surfaces. Bones were considered as rigid bodies. The Anterior cruciate ligament (ACL), posterior cruciate ligament (PCL), and medial and lateral collateral ligaments (MCL, LCL) were modelled as bi-linear springs that could withstand tension but not compression. Tensile stiffness k = 380 N/mm was used for the ACL^50^, whereas k = 200 N/mm was used for the PCL^51^. Tensile stiffness for the MCL and LCL were k = 100 N/mm^51,52^.

The middle-central position between the medial and lateral epicondyles of the femur was used as the reference point for coupling the femur surface to the loading^53,54^. The bottom nodes of the tibial cartilage were fixed. The cartilage surfaces at the calcified zone were impermeable, while the pore pressure of the articular cartilage surfaces was set to zero, permitting free fluid flow. A gait stance phase was simulated by applying a combination of an indentation load and a flexion angle at the reference point (Fig. 1)^53,55^. A settling step was considered before the stance phase, where a load of 30 N was applied for one second on the reference point of the femur to make the initial contact of cartilage surfaces. Abaqus/CAE software 2018 (Dassault Systems Simulia Corp., Johnston, RI, USA) was used for the FE modeling. The FE mesh was done in HyperMesh 2019 (Altair Inc, Santa Ana, CA).

### Evaluation metrics for segmentation

The metrics to evaluate the segmentation performance of the femur and tibia using the Swin UNETR and SSM methods included the DSC, Hausdorff distance, average distance, and the percentage of surface area associated with a distance greater than 1 mm between the two methods. The DSC measures the overlap between the segmented regions and the manual segmentation as the ground truth (intersection over union). The Hausdorff distance quantifies the maximum distance from the nearest neighbor^56^ between corresponding points on the segmented surface and the ground truth. The other calculated parameter to assess the accuracy of the semi-automated method was the average distance, which represents the average separation between the segmented surface and the ground truth surface. The percentage of surface area associated with a distance greater than 1 mm (∆area% > 1mm) represents the percentage of surface area where the distance between the segmented regions and the ground truth exceeds 1 mm.

### Statistical analysis for FE

To compare the mechanical response from the manual and semi-automated FE models, 5 parameters, including the max principal stress, max principal strain, fluid pressure, fibril strain, and contact area, were considered for the duration of a stance simulation. The first 4 parameters were compared in superficial and deep zones, while the contact area was only calculated on the articular surface of the cartilage. In each zone, the average and peak values of these parameters were compared. We selected the statistical parametric mapping (SPM) method based on its inherent advantage in accommodating multiple comparisons when examining smooth and random 1-D trajectories. In contrast to traditional 0-D approaches, such as the parametric t-test, the SPM method demonstrates superior suitability for this purpose^57^. The SPM t-test was performed for two independent samples with a criterion alpha-level of 0.05. The SPM was implemented using a Python package from https://spm1d.org/# for 1-D SPM.

## Results

Table 1 presents the evaluation metrics for the segmentation performance of the femur and tibia using the Swin UNETR and SSM methods. For all bone structures and segmentation methods, the DSC was consistently high, with a value over 98%. The Swin UNETR method achieved a Hausdorff distance of 1.66 ± 0.34 mm for femur and 1.65 ± 0.48 mm for tibia. The SSM adjustment resulted in a slightly lower Hausdorff distance of 1.42 ± 0.37 mm for femur and 1.47 ± 0.41 mm for tibia. For the femur and tibia, the Swin UNETR method resulted in an average distance of 0.30 ± 0.04 mm and 0.31 ± 0.03 mm, with the SSM adjustment yielding a slightly lower value of 0.23 ± 0.05 mm and 0.25 ± 0.043 mm, respectively. The femur and tibia segmentation using the Swin UNETR method showed a ∆area% > 1 mm of 0.98 ± 1.61% and 1.11 ± 1.25%, respectively. After the SSM adjustment, the ∆area% > 1 mm values slightly decreased to 0.57 ± 1.10% and 0.71 ± 1.01%, respectively. Figure 3S in the supplementary material illustrates a comparison of manual and Swin UNETR segmentations. The semi-automated segmentation took approximately 10 min of computational time (5 min for Swin UNETR and 5 min for SSM) to produce tibia and femur geometry as compared to the manual segmentation, which took about 2 h from an expert to segment tibia and femur (~ 90 min for the manual segmentation in ITK-SNAP software, ~ 30 min for smoothing the model in MeshLab). A video was recorded on how to implement the Swin UNETR model at https://data.mendeley.com/datasets/k5hdc9cz7w/1.

**Table 1:** DSC, Hausdorff distance (mm), average distance (mm), and percentage of surface area associated with a distance greater than 1 mm for Swin UNETR and SSM.

	DSC	Hausdorff distance (mm)	Average distance (mm)	$\Delta area\%>1 {\text{mm}}$
Femur Swin UNETR	98.51 ± 0.09	1.66 ± 0.34	0.30 ± 0.04	0.98 ± 1.61
Tibia Swin UNETR	98.59 ± 0.06	1.65 ± 0.48	0.31 ± 0.03	1.11 ± 1.25
Femur SSM adjustment	98.63 ± 0.11	1.42 ± 0.37	0.23 ± 0.05	0.57 ± 1.10
Tibia SSM adjustment	98.69 ± 0.07	1.47 ± 0.41	0.25 ± 0.04	0.71 ± 1.01

**Figure 4:**
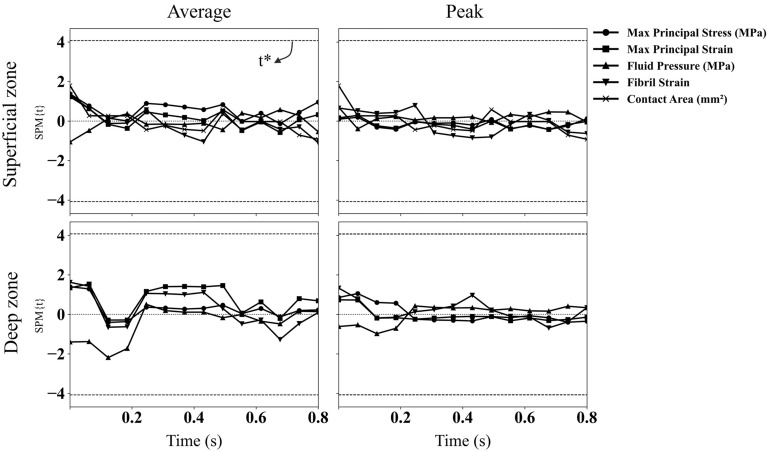
Statistical parametric mapping (SPM) as a function of time for the mechanical response of 5 parameters in superficial and deep zones. The dashed line shows the t-critical corresponding to a p-value of 0.05. The average column shows the average of the respected parameter over the contact region of all samples. Similarly, the Peak column shows the maximum value of these 5 parameters. The contact region over the articular surface was projected into the deep zone to calculate parameters in the deep zone.

Statistical analysis showed no significant difference between the manual and semi-automated FE models for all 9 samples. Figure 4 depicts the SPM of maximum principal stress and strain, fluid pressure, fibril strain, and contact area as a function of time (s). All parameters were within the critical values indicating no significant difference (*p*-value > 0.05).

**Figure 5:**
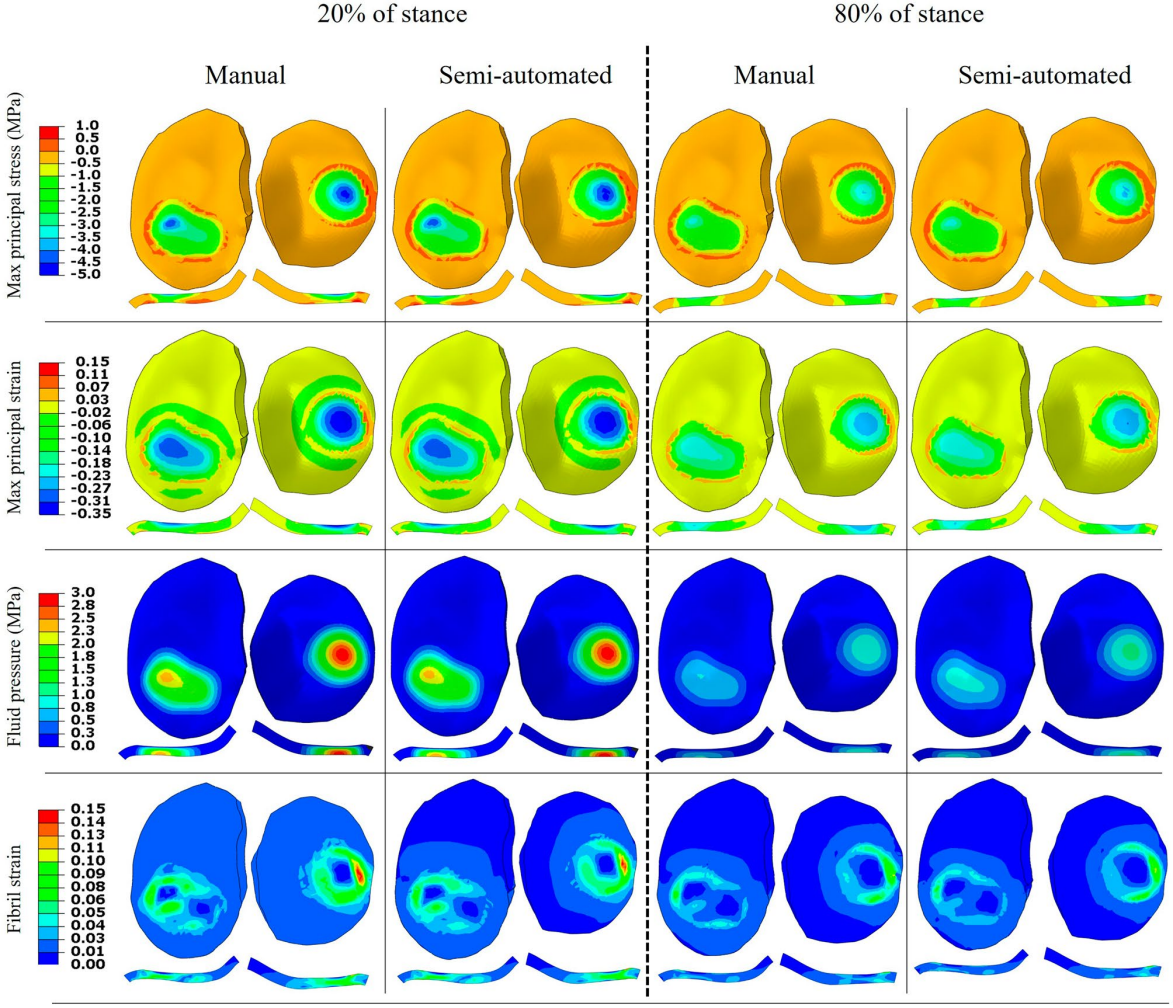
The distribution of maximum principal stress and strain, fluid pressure, and fibril strain over the surface and thickness of the tibial compartments at 20% and 80% of the stance phase for subject 2. The depth-wise illustration was from the cross-section where the peak value occurred.

The distribution of mechanical responses over the surface and depth-wise at 20% and 80% of the stance phase were illustrated in Fig. 5 for subject 2. The manual and semi-automated FE models resulted in a similar distribution of parameters. Figures 4S–7S in the supplementary material illustrate the distribution for each sample in tibial and femoral cartilages for each of the five mechanical responses.

**Figure 6:**
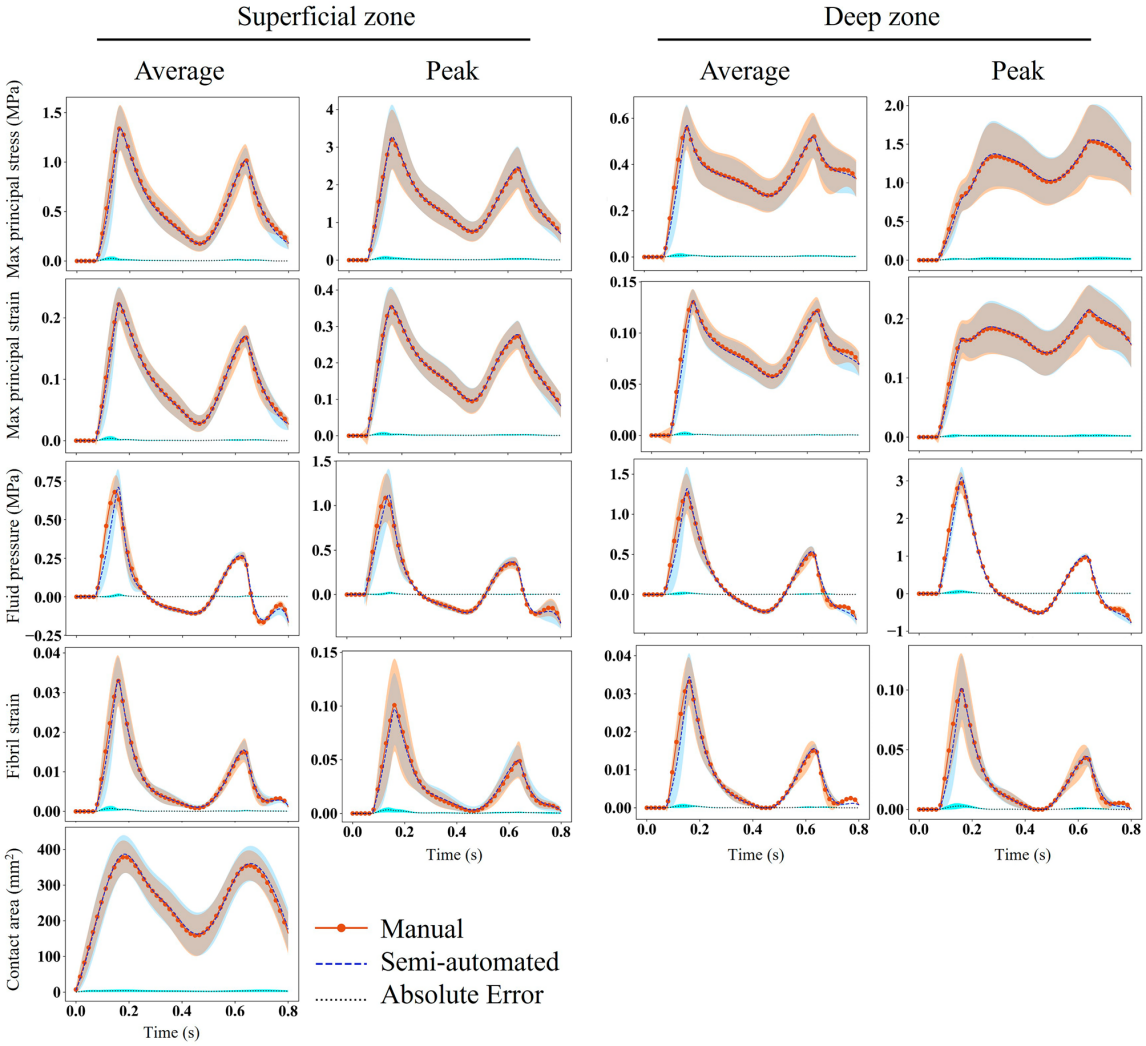
The average (over contact area) and maximum values of maximum principal stress and strain, fluid pressure, and fibril strain in the superficial and deep zones. The shaded region represents one standard deviation. The solid line with a circular marker represents the manual FE model, whereas the dashed line represents the semi-automated FE model. The dotted line is the absolute difference between the two models. The contact region over the articular surface of the cartilage was projected into the deep zone to calculate the parameters.

The average and peak values of the mechanical parameters in the superficial and deep zones are illustrated in Fig. 6. The dotted line represents the absolute differences between the two FE models. The contact region over the articular surface was projected into the deep zone to measure the mechanical parameters in the deep zone in Fig. 6. The fluid pressure had the largest error of 0.01 MPa. In the supplementary material, Figs. 8S–11S are plotted for each sample separately to provide a more detailed comparison between the semi-automated and manual FE models. All these figures indicated no significant variation in the mechanical response of the semi-automated FE model compared to the manual FE model.

## Discussion

**Figure 7:**
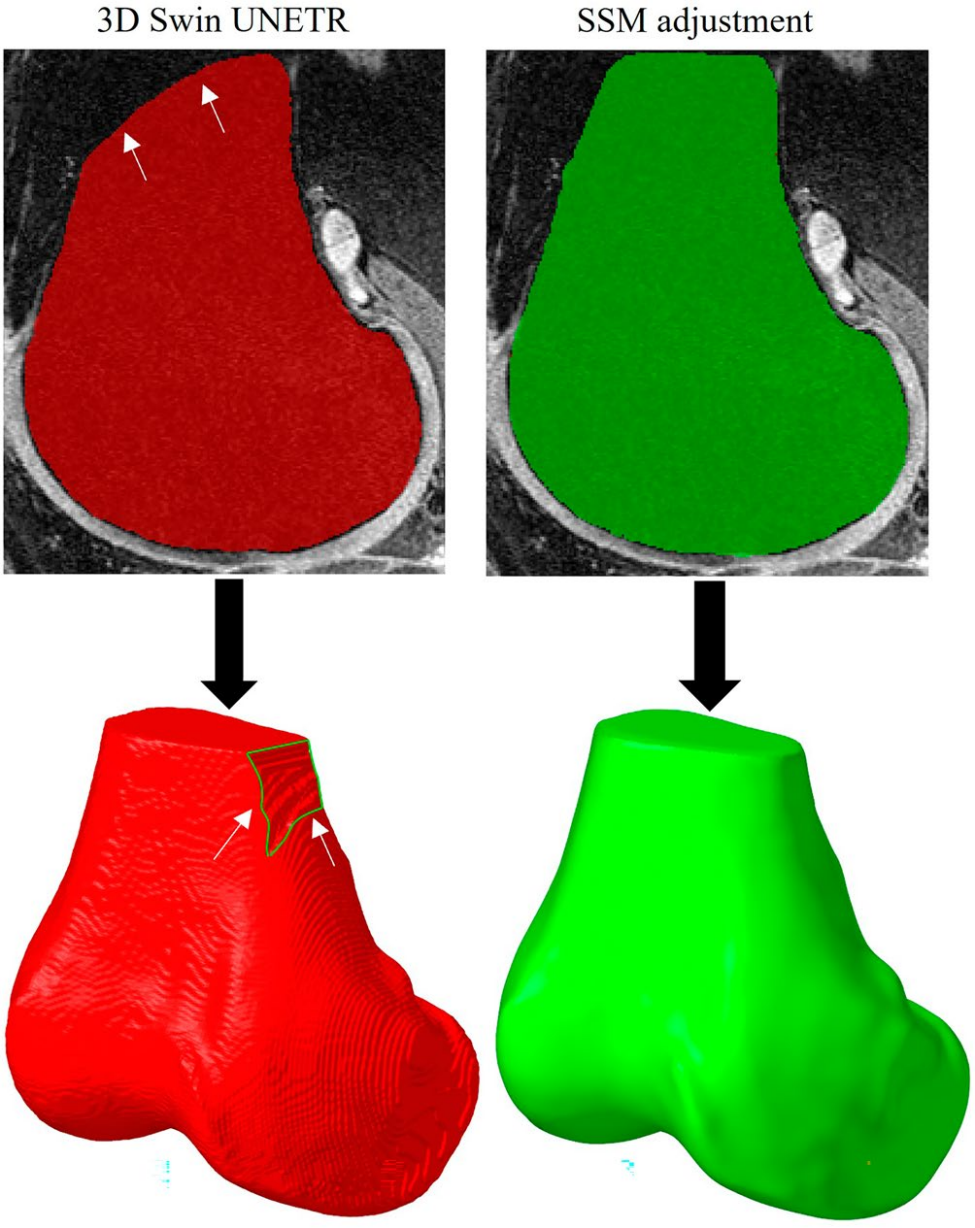
The outcome of the 3D Swin UNETR has a coarse surface topology with occasional artifacts, noises and holes (shown by arrows) that require post-processing before being used in a FE study. The SSM adjustment produced high-quality surfaces without compromising the accuracy of the segmentation.

In the present study, a trained SSM model of tibia and femur was mapped to a Swin UNETR segmentation model. The Swin UNETR generated a personalized geometry from MRIs, and the SSM automated the post-processing operations associated with filing holes and smoothing surfaces, which are essential steps to increase the convergency rate in FE simulations (Fig. 7)^22,25,58^. By incorporating prior knowledge and capturing shape variations from a training dataset of 507 MRIs, the SSM adjustment consistently delivered high-quality surfaces in the context of image segmentation. These benefits make the proposed Swin UNETR and SSM a valuable semi-automated approach for accurate and robust FE model development from the tibia and femur MRIs.

Generally, geometrical models, such as SSM, require manual landmark selection by the user. This can negatively impact the accuracy, reproducibility, and segmentation time due to intra-individual variability. However, we tackled this challenge by employing Swin UNETR to generate unlimited anatomical landmarks automatically for SSM. Such models can capture spatial dependencies and long-range context information, leading to more precise segmentations^25^.

Overall, the segmentation performance of the Swin UNETR model and SSM adjustment exhibited high DSC values (Table 1), indicating a strong agreement with the ground truth (i.e., the manual segmentation). The combination of Swin UNETR and SSM methods demonstrated lower Hausdorff distances and lower average distances compared to the Swin UNETR method, indicating better boundary conformity and closer agreement with the ground truth surface. Furthermore, the ∆area% > 1mm values indicated minimal discrepancies in the segmented surface area for both methods (Table 1). In comparison to existing algorithms for segmenting knee images, the DSCs in our research were in the range of 98.6% for femur and 98.7% for tibia. These results are on par with the performance of previous works. For instance, a recent study reported DSC of 96.2% for tibia and 97.0% for femur^59^. Two other recent studies obtained DSC of 98–99% for tibia and 98.6 for femur^24,25,60^. However, these studies did not use their segmentation model in biomechanics (under physical loading for mechanical responses). Our study attempted to bridge the gap between a highly advanced segmentation model and its application in biomechanical engineering. This is essential given that a high DSC does not guarantee a suitable shape for mechanical modeling; a shape must be tailored so that proper meshing and interactions can be made feasible in FE modeling.

The 20% and 80% stance phase selected for evaluating the most common mechanical metrics^11,15,16,54,55^ in Fig. 5 corresponded to the two peaks of the loading condition (Fig. 1). A strong agreement was found for the distribution of all parameters between the two models, except for fluid pressure, for which the semi-automated FE model resulted in a larger fluid pressure at 80% stance phase compared to the manual FE model. This was reflected in a larger t-value of SPM for fluid pressure compared to the other four parameters (Fig. 4); however, the respected values were well below the t-critical value and thus, the fluid pressure difference between the manual and semi-automated FE models was not significant.

Figure 5 illustrates a qualitative comparison of the mechanical responses between the manual and semi-automated FE models for one sample at 20% and 80% of the stance phase, while Fig. 6 provides quantitative comparisons over the entire stance phase averaged for elements in the contact region, where the five parameters had higher magnitudes across the model. The time scale in Fig. 6 corresponds to the one used in statistical analysis (SPM) presented in Fig. 4. From the analysis of these figures, it becomes evident that despite some discrepancies, there were no significant differences (*p*-value > 0.05) between the manual and semi-automated FE models across the entire stance phase and samples. These results highlight the reliability and accuracy of the semi-automated segmentation approach, supporting its potential as a viable alternative to manual segmentation for the analysis of mechanical properties in the studied samples.

Multiple factors unrelated to the semi-automated segmentation method may affect the variation in mechanical responses when comparing two FE models. For instance, model outputs are available at discrete time points selected by the FEA solver at each time increment of the analysis. These time points vary slightly from one model to another. For instance, the maximum principal stress and strain distributions in Fig. 5 were plotted at 19.91% and 20.14% of the stance phase for manual and semi-automated methods, respectively. That is because those time points were the closest to the 20% stance. A high temporal resolution can rectify this issue. Nevertheless, the temporal resolution effect may become more pronounced for FE models with high loading rates, especially when the comparison is conducted at the time instances where applied loads are at the peak. The other source of variations in the results of FE models is spatial resolution. Mechanical responses of FE models are available at element nodes or element Gaussian points. When meshing the geometry, it may not be possible to generate an identical mesh for the two models due to differences in geometry. Therefore, the location of two corresponding points used for comparison may vary slightly from one model to another. Moreover, there was no correlation between the Hausdorff distance and the mechanical error. For example, subjects 3 and 6 had relatively larger errors than other subjects (Figs. 8S–11S in the supplementary material), but their corresponding Hausdorff distances were below and above, respectively, the average values in Table 1. Likewise, we could not attribute errors in mechanical parameters to the differences in the OA severity. The KL grade and Hausdorff distance vs. mechanical responses of 9 models in Figs. 12S in the supplementary material, had correlation coefficients R^2^ in the range of 0.01–0.2. Nevertheless, this conclusion is drawn from the examination of 9 FE models in this study, and it may be subject to change with a larger dataset.

The current study has some limitations. One limitation of our study was the exclusion of the meniscus and cartilage contact in the finite element modeling of knee cartilage^61,62^; however, in the context of our specific research objectives and scope, this omission does not significantly impact the findings and conclusions drawn. While the meniscus and cartilage contact play important roles in knee biomechanics, their inclusion would have significantly increased the computational time for FE modeling. Given the focus and objectives of our research, the decision to exclude these components does not compromise the validity and relevance of our study findings^16^. Another limitation of our study is the small number of samples used in FE modeling, which may limit the generalizability of our findings. We included nine samples for finite element modeling. Future studies should aim to include a larger sample size and consider the use of automatic meshing techniques^20,63^, while ensuring consistent and reliable geometries to achieve a high convergence rate^57^. Additionally, the spring elements representing the ligaments did not include the wrapping effect of ligaments. This might affect the FE element outcome^64^. However, since we have considered the same simplifications for both models, neglecting the wrapping effect of ligaments would not likely affect the interpretation of results in the present study. Lastly, the cartilage geometry was segmented, and ligaments' insertion points were labeled manually from MRIs for both manual and semi-automated models. This is to avoid multifactorial effects and assess the performance of the semi-automated bone segmentation model. In future studies, the presented procedure can be applied to cartilage, instead of manual segmentation, along with an automatic cartilage mesh generation technique^65^. This will advance the development of the knee joint FE model towards full automation.

In summary, the integration of Swin UNETR and SSM has demonstrated remarkable effectiveness in the segmentation of MRIs. By harnessing the strengths of both Swin UNETR and SSM, this method not only enhances segmentation precision but also creates suitable shapes and geometries for FE models. We have released our semi-automated segmentation models to the public (https://data.mendeley.com/datasets/k5hdc9cz7w/1), aiming to contribute to the progress of biomechanical modeling and medical image segmentation. The ultimate goal of this study is to develop a segmentation of knee joint components, including cartilage, ligaments, and meniscus, to further facilitate computational modeling. This will help the biomechanical community swiftly achieve subject-specific knee joint segmentation.

### Supplementary Information

Supplementary Information.

## Data Availability

Please refer to the https://data.mendeley.com/datasets/k5hdc9cz7w/1 for segmentation models. For OAI please refer to https://nda.nih.gov/oai/ (please email the website to request the images). FE models are available upon request to Reza Kakavand at reza.kakavand@ucalgary.ca.
