# Supplementary material for: Integration of Swin UNETR and statistical shape modeling for a semi-automated segmentation of the knee and biomechanical modeling of articular cartilage
Source: Sci Rep. 2024 Feb 2;14:2748. doi: 10.1038/s41598-024-52548-9 (PMC10834430; doi:10.1038/s41598-024-52548-9)
Supplement: Supplementary file 1 — Supplementary Information. [file 41598_2024_52548_MOESM1_ESM.docx]

***Supplementary Material:***

Integration of Swin UNETR and statistical shape modeling for a semi-automated segmentation of the knee and biomechanical modeling of articular cartilage

Reza Kakavand^1^, Mehrdad Palizi^2^, Peyman Tahghighi^1^, Reza Ahmadi^1^, Neha Gianchandani^1^, Samer Adeeb^2^, Roberto Souza^3,4^, W. Brent Edwards^1^, Amin Komeili^1*^

^1^Department of Biomedical Engineering, Schulich School of Engineering, University of Calgary

^2^Civil and Environmental Engineering Department, Faculty of Engineering, University of Alberta

^3^Department of Electrical and Software Engineering, Schulich School of Engineering, University of Calgary

^4^Hotchkiss Brain Institute, Cumming School of Medicine, University of Calgary

**Corresponding author: Amin Komeili**

***** [**amin.komeili@ucalgary.ca**](mailto:amin.komeili@ucalgary.ca) **Address: ICT243, 2500 University Drive NW, Calgary, AB, T2N 1N4**

The matrix and fibril stresses were determined using energy functions:

| $\sigma= -pI+\emptyset_{0}J^{-1}F \left( 2\frac{\partial W_{0}\left( C \right)}{\partial C} \right)F^{T}+\emptyset_{1}J^{-1}F\left( 2\frac{\partial W_{1i}(C)}{\partial C}+2\frac{\partial\bar{W}_{1a}(\bar{C})}{\partial C} \right)F^{T}$ | (2) |
| --- | --- |

where *J* is the determinant of *F* (deformation gradient), $\bar{C}$ is the distortional component of the right Cauchy-Green deformation tensor, *C.* $W_{0}$ and $W_{1i}$ are the Holmes-Mow (Holmes and Mow, 1990) elastic strain energy potential of isotropic matrix and collagen fibrils, respectively, defined as:

| $W_{HM}\left( C \right)=\alpha_{0}\frac{exp[\alpha_{1}\left( I_{1}\left( C \right)-3 \right)+\alpha_{2}{(I}_{2}\left( C \right)-3)]}{{{(I}_{3}\left( C \right))}^{\beta}}$ | (3) |
| --- | --- |

Where $\alpha_{0}$, $\alpha_{1}$, $\alpha_{2}$ and $\beta$ are the material constants. $\bar{W}_{1a}$ was expressed as a function of the distortional component of *C,* because the collagen fibrils were assumed incompressible (Federico and Gasser, 2010; Komeili et al., 2020):

| $\bar{W}_{1a}\left( \bar{C} \right)=\int_{S_{X}^{2}} \psi(\vec{M})\times\frac{1}{2}c_{1b}\left[ \bar{I}_{4}\left( \bar{C},A\left( \vec{M} \right) \right)-1 \right]^{2}dS$ | (4) |
| --- | --- |

where $c_{1b}$ is a material constant, $\bar{I}_{4}$ is the fourth invariant of $\bar{C}$*,* $A\left( \vec{M} \right)=\vec{M}\otimes\vec{M}$ is a structure tensor that is a function of fibrils direction in the reference configuration$(\vec{M})$. The $\psi(\vec{M})$ is a probability distribution density function that gives the probability of finding a fibril aligned with the direction $\vec{M}$(Federico and Gasser, 2010):

| $\psi\left( \vec{M} \right)=\rho\left( \Theta\right)=\frac{1}{\pi}\sqrt{\frac{b}{2\pi}}\frac{exp[b\left( \cos2\Theta\right)+1]}{erfi(\sqrt{2b})}$ | (5) |
| --- | --- |

Where $\Theta$ is the co-latitude in polar coordinates, *erfi*(*x*) is the imaginary error function and the direction of the fibrils are controlled by the parameter b; negative and positive values of b produce parallel and perpendicular to surface fibrils orientation, while b=0 generates equally distribute fibrils over a sphere, i.e. random fibers distribution. Table 1 provides a description of the biphasic model and associated material constants.

Table 1S. Material constants of the model (Komeili et al., 2020).

| Material properties | | Collagen Fibril | |  | ECM | |
| --- | --- | --- | --- | --- | --- | --- |
|  |  | SZ | DZ |  | SZ | DZ |
| E (MPa)† |  | 10 | 15 |  | 2.5 | 3.8 |
| *ν* † |  | 0.3 | 0.3 |  | 0.1 | 0.1 |
| $\alpha_{0}$† |  | 3.4 | 5.1 |  | 0.6 | 1.0 |
| $\alpha_{1}$† |  | 0.1 | 0.1 |  | 0.8 | 0.8 |
| $\alpha_{2}$† |  | 0.4 | 0.4 |  | 0.1 | 0.1 |
| $c_{1b}$† (MPa) |  | 7.6 | 11.4 |  | _ | _ |
| *β* † |  | 1.0 | 1.0 |  | 1.0 | 1.0 |
| *k* † |  | _ | _ |  | 2.8 | 2.8 |
| e_R_* † |  | _ | _ |  | 4.0 | 4.0 |
| b †† |  | 0 | 0 |  | 0 | 0 |
| Thickness‡ |  | 0.12h | 0.62h |  | 0.12h | 0.62h |
| * Void ratio (fluid / solid volume)  SZ: Superficial Zone  DZ: Deep Zone  h: Cartilage thickness  † (Pajerski, 2010)  †† (Komeili et al., 2020)  ‡ (Julkunen et al., 2007) | | | | | | |

Figure 1S illustrates the data distribution through the five-fold cross-validation for training and testing the Swin UNTR model.

Figure 2S represents the workflow of developing FE models of the cartilage and femur bone, including surface preparation, geometry partitioning, mesh generation, and assembly components.

Figure 3S presents the results of the MRI segmentation comparison between the manual segmentation and the Swin UNETR segmentation method. The purpose of this evaluation is to visually assess the performance of the Swin UNETR model in segmenting MRI and to compare it against the gold standard of manual segmentation. The green contour overlaid on the Swin UNETR segmentation indicates the alignment with the outline obtained from manual segmentation.

The contour plots in Figure 4S depict the distribution of maximum principal stress over the surface and along the cartilage thickness of all nine pairs of manual and auto-segmentation models at 20% of the stance phase. Similarly, Figure 5S shows the distribution of the maximum principal strain, Figure 6S demonstrates the fluid pressure distribution, and Figure 7S showcases the distribution of fibril strain. The x-y cross-section includes the point with the maximum value of the depicted variable in the corresponding figure. This is to ensure that the comparison is made where the largest error is expected.

The average and peak values of mechanical parameters in superficial and deep zones for all FE models are depicted in Figure 8S to Figure 11S. In these figures, solid and dashed lines represent the manual and semi-automated models, respectively. The dotted line illustrates the absolute difference between the two models. It is important to note that the average values were calculated over the contact region, and the peak values reflect the maximum value of the paramter in the cartilage. Additionally, the contact region of the superficial zone was projected into the deep zone for calculating parameters in that region.


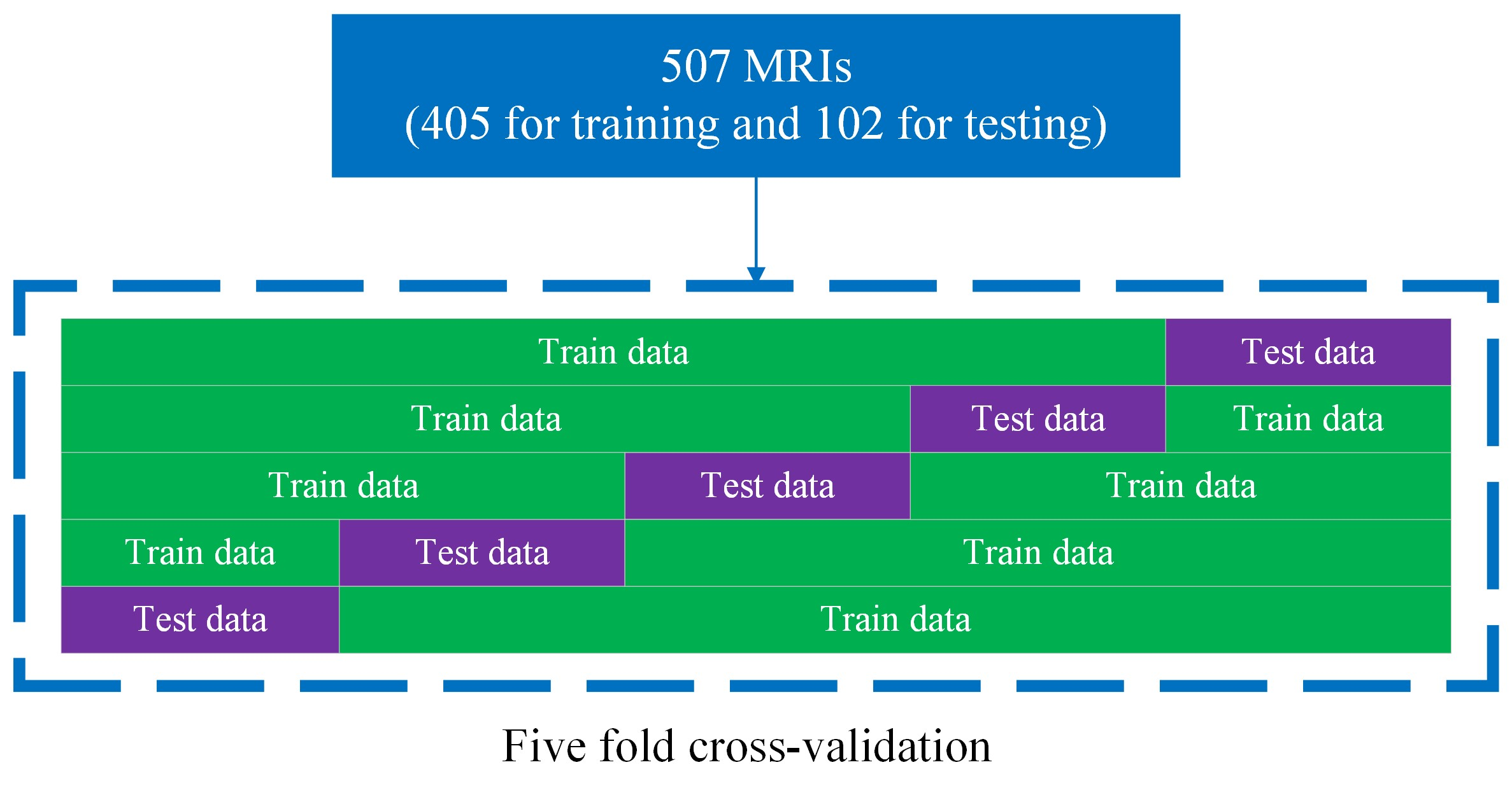


Figure 1S. Five-fold cross-validation was used for training Swin UNETR and SSM models in our study.


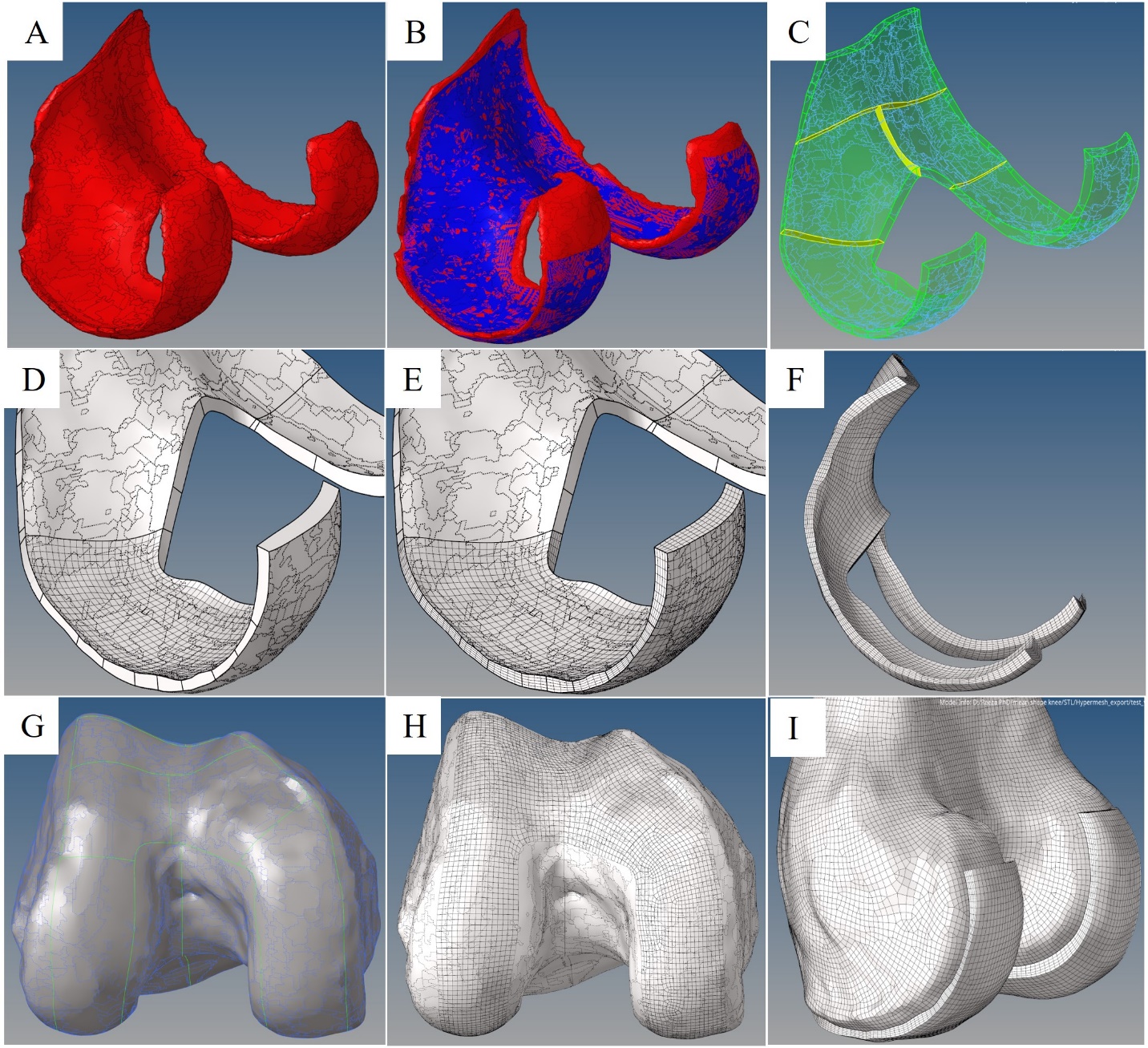


Figure 2S. Mesh generation: (A) Cartilage surfaces were imported to HyperMesh as 2D triangular elements, which were converted to an enclosed volume (using geometry/create/surface/from FE) and then were converted to a solid. (B) The jagged boundary of the cartilage perimeter was trimmed to facilitate 3D mesh generation. (C) The geometry was partitioned into different sections, giving more control over the mesh. (D) For each partition, the face that is shared between the cartilage and calcified zone was meshed using the "2D automesh/ batchmesh QI optimize". (E) The "3D solid map/ one volume" was used to map the 2D mesh of the calcified zone to the articular surface and create depth-wise element size. (F) A fully-meshed cartilage model was illustrated. (G) The intersection surface of the cartilage and calcified zone was used to partition the bone top surface (HyperMesh/ Geometry/ surface edit/ trim with nodes). (H) this partition was meshed using "2D automesh/ batchmesh QI optimize". (I) The complete femoral FE model.


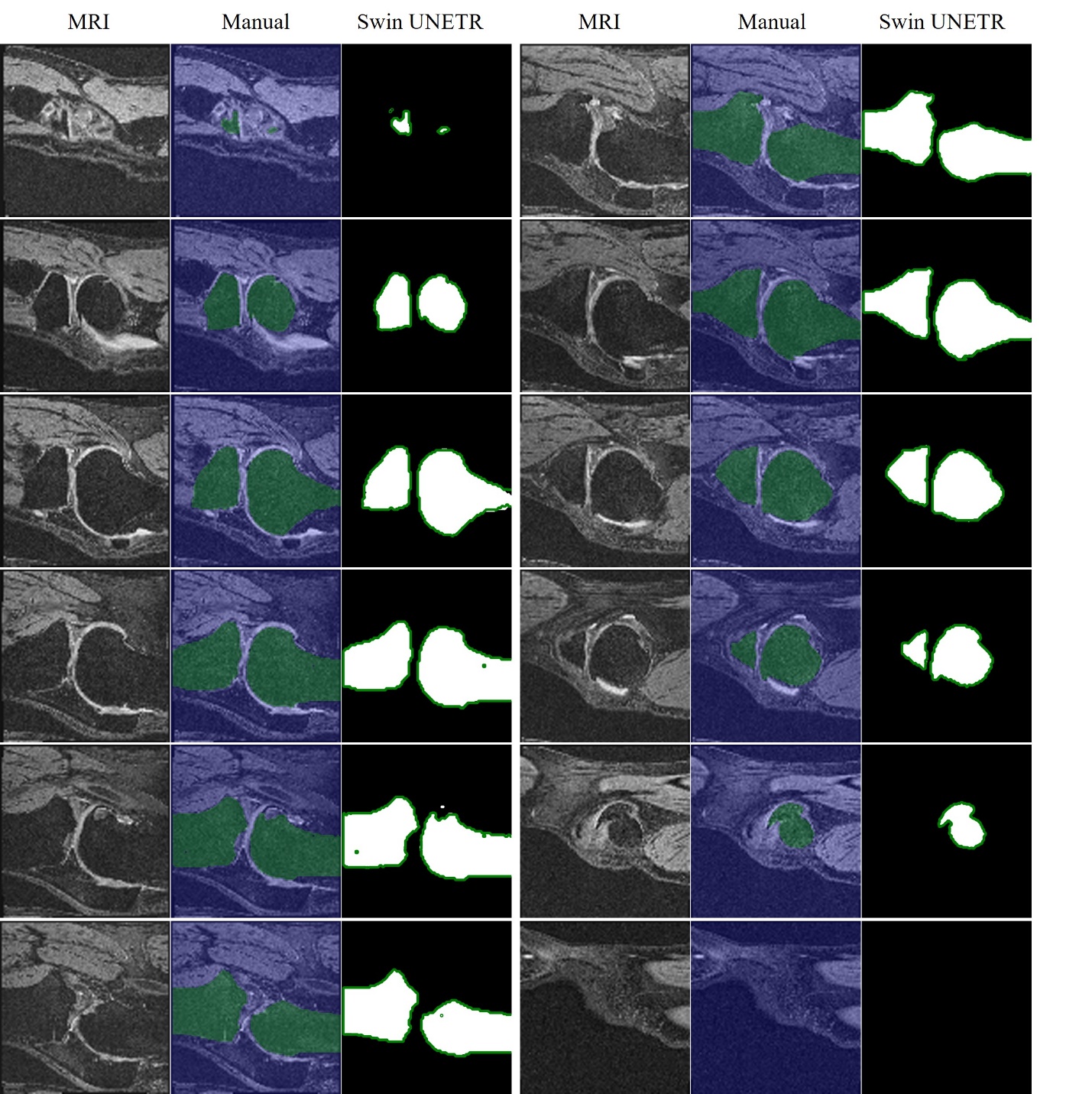


Figure 3S. A comparison of MRI segmentation results using the manual segmentation and Swin UNETR segmentation. The green contour on Swin UNETR segmentation images is the outline from the manual segmentation.


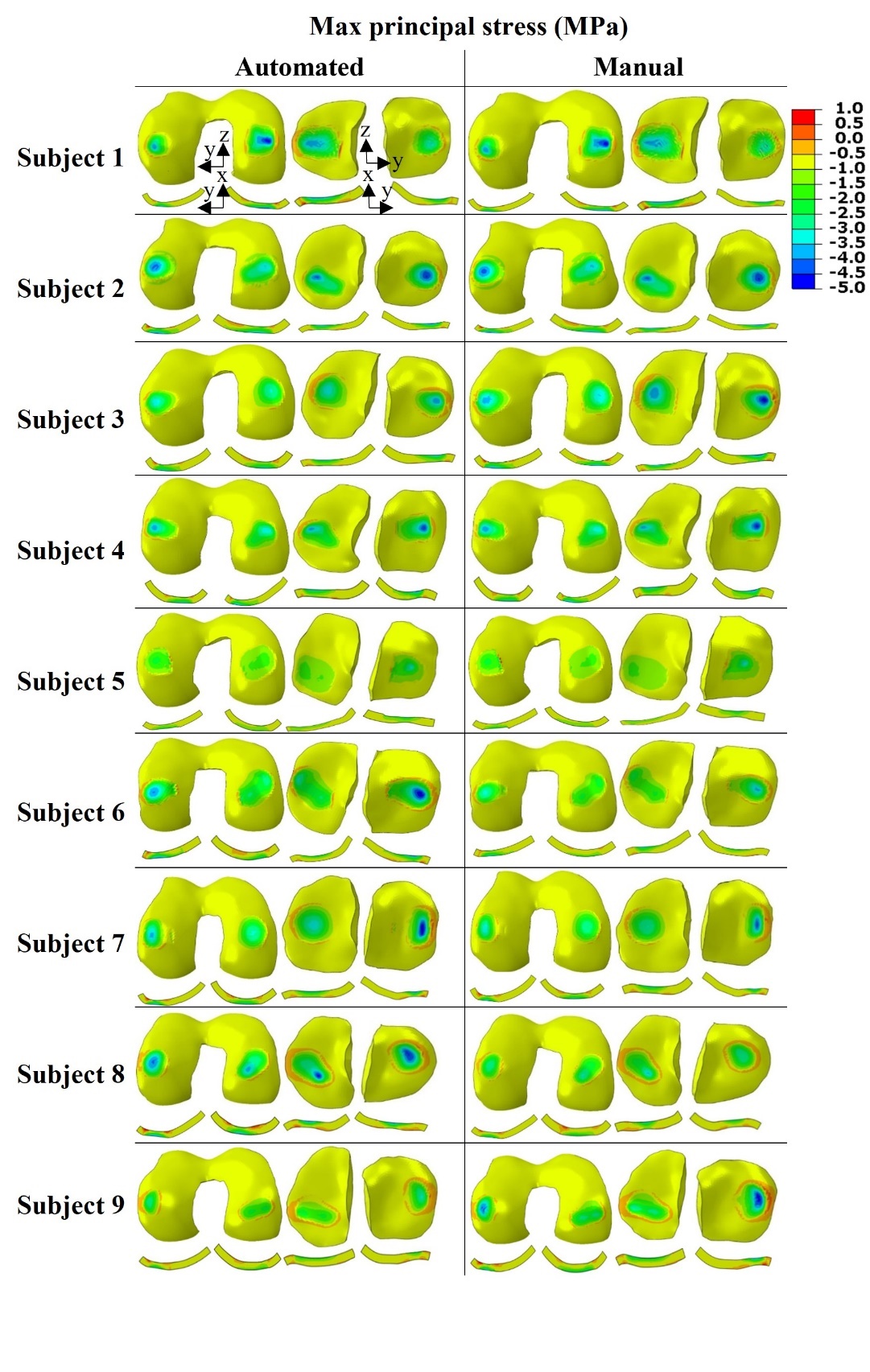


Figure 4S. The distribution of maximum principal stress over the surface and along the thickness of nine cartilage models at 20% of the stance phase. The depth-wise illustration was from the cross-section where the peak value occurred.


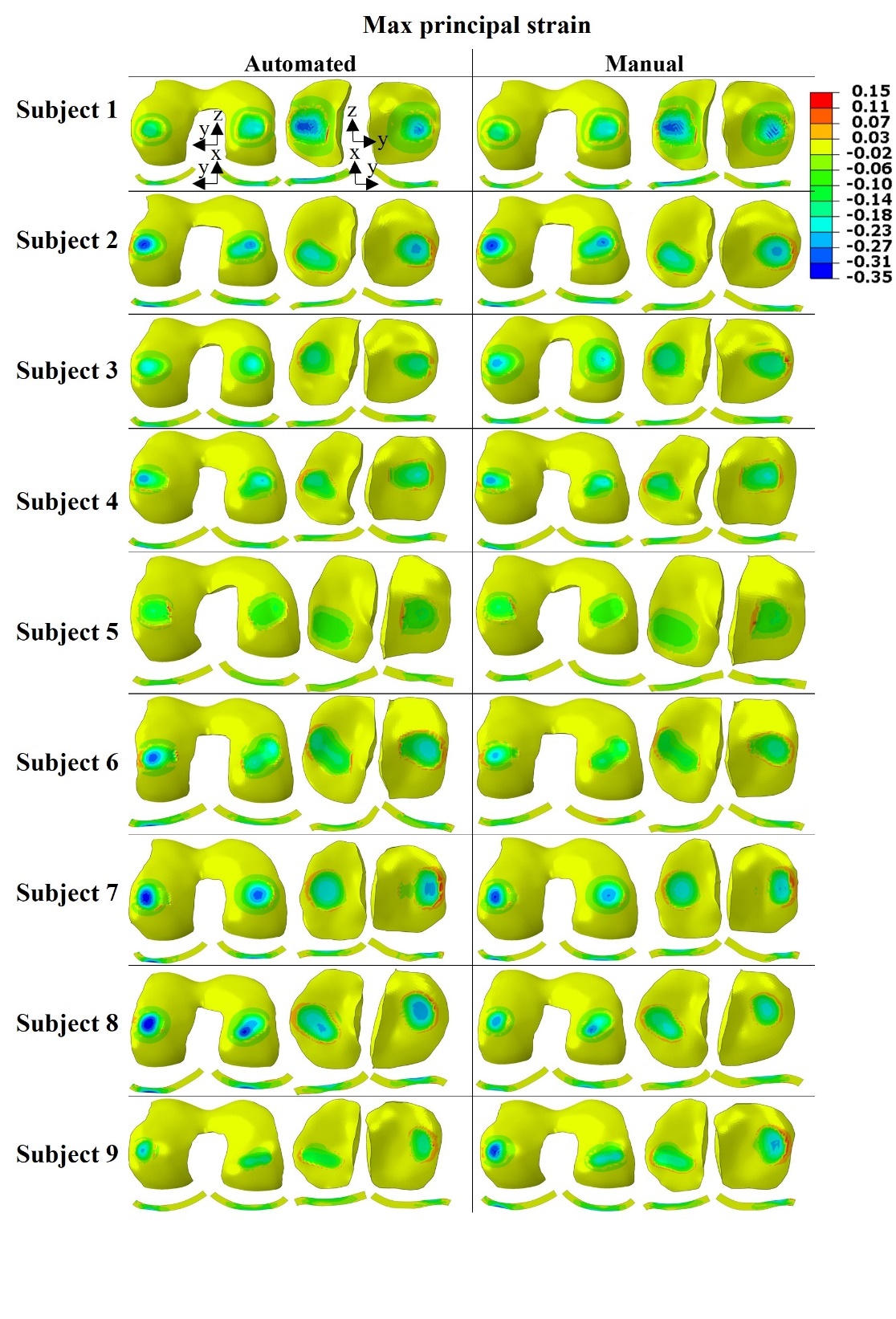


Figure 5S. The distribution of maximum principal strain over the surface and along the thickness of nine cartilage models at 20% of the stance phase. The depth-wise illustration was from the cross-section where the peak value occurred.


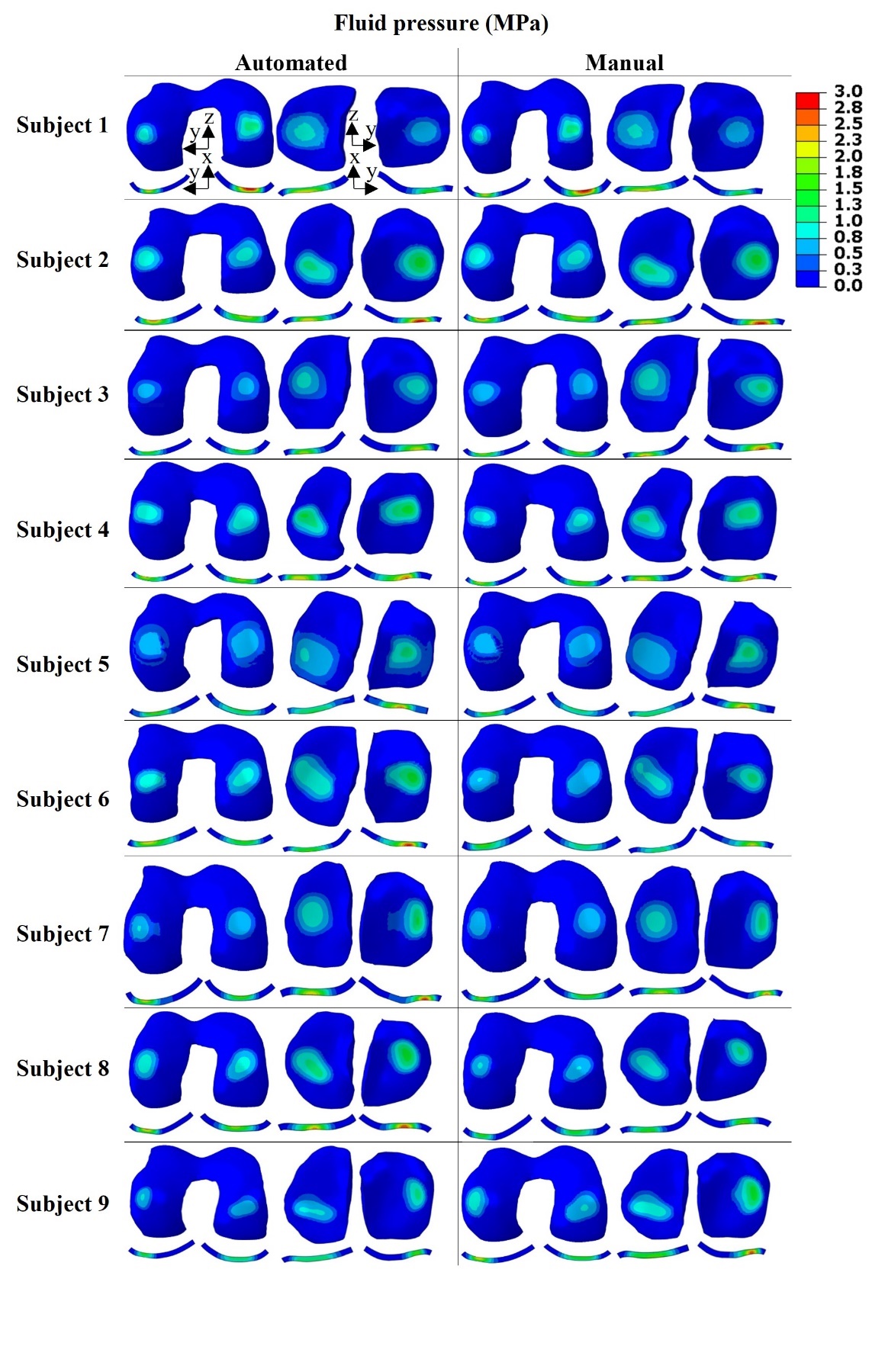


Figure 6S. The distribution of fluid pressure in the superficial zone and along the thickness of nine cartilage models at 20% of the stance phase. The depth-wise illustration was from the cross-section where the peak value occurred.


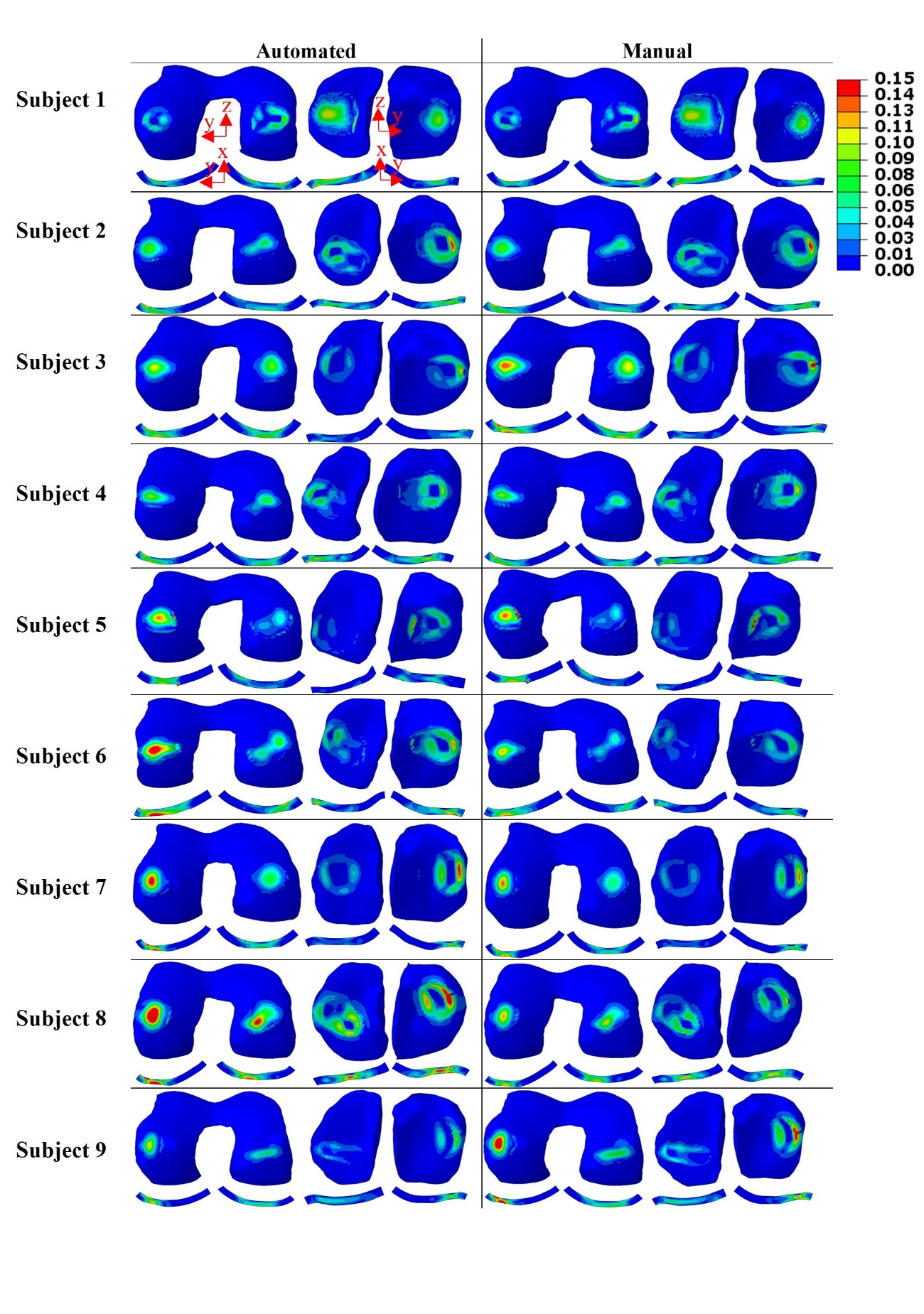


Figure 7S. The distribution of fibril strain in over the surface and along the thickness of nine cartilage models at 20% of the stance phase. The depth-wise illustration was from the cross-section where the peak value occurred.


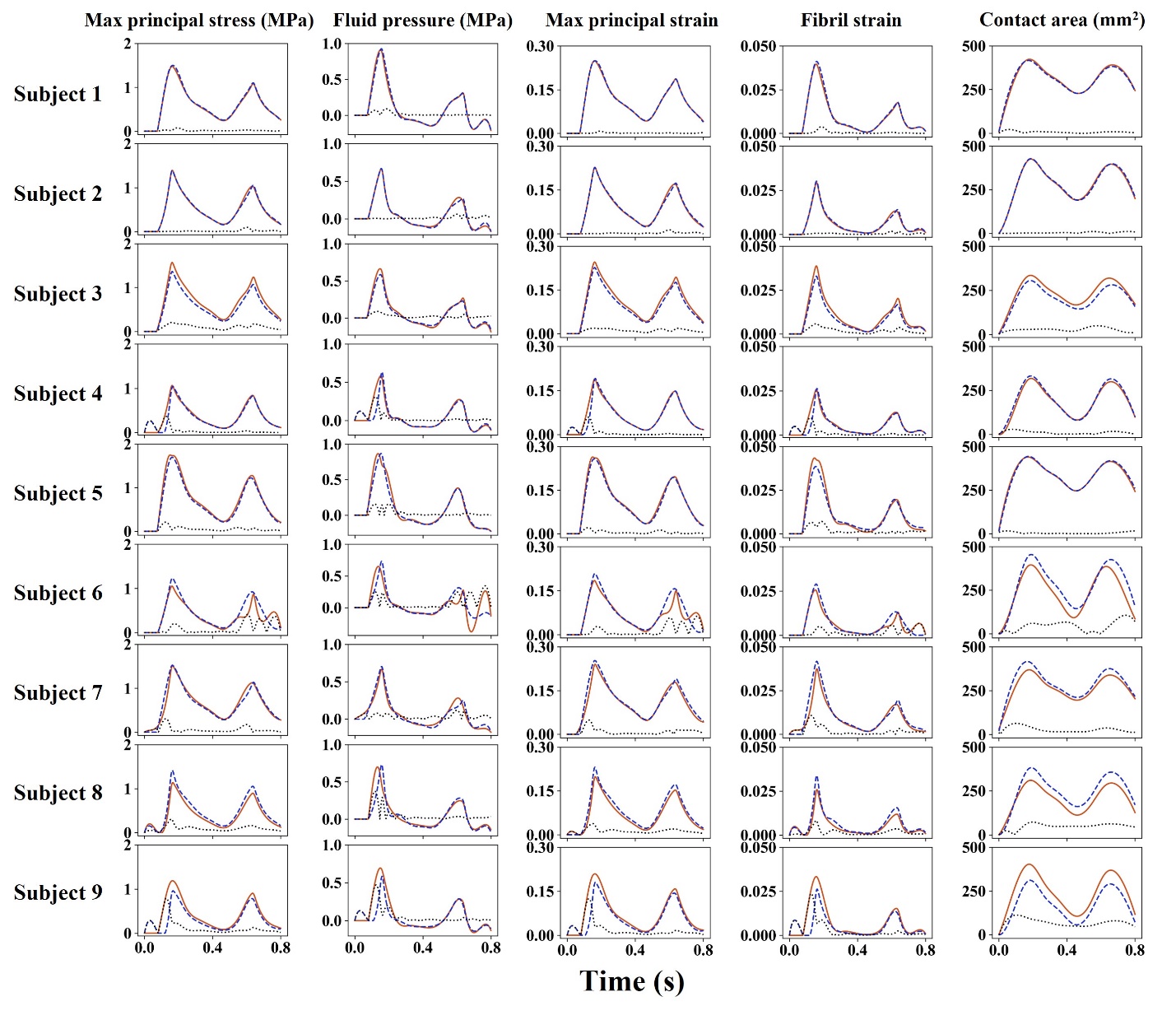


Figure 8S. The **average** values of the mechanical parameters in the **superficial zone** for all cartilage models. The solid and dashed lines represent the manual and semi-automated models, respectively. The dotted line is the absolute difference between the two models. Values were calculated from the contact region.


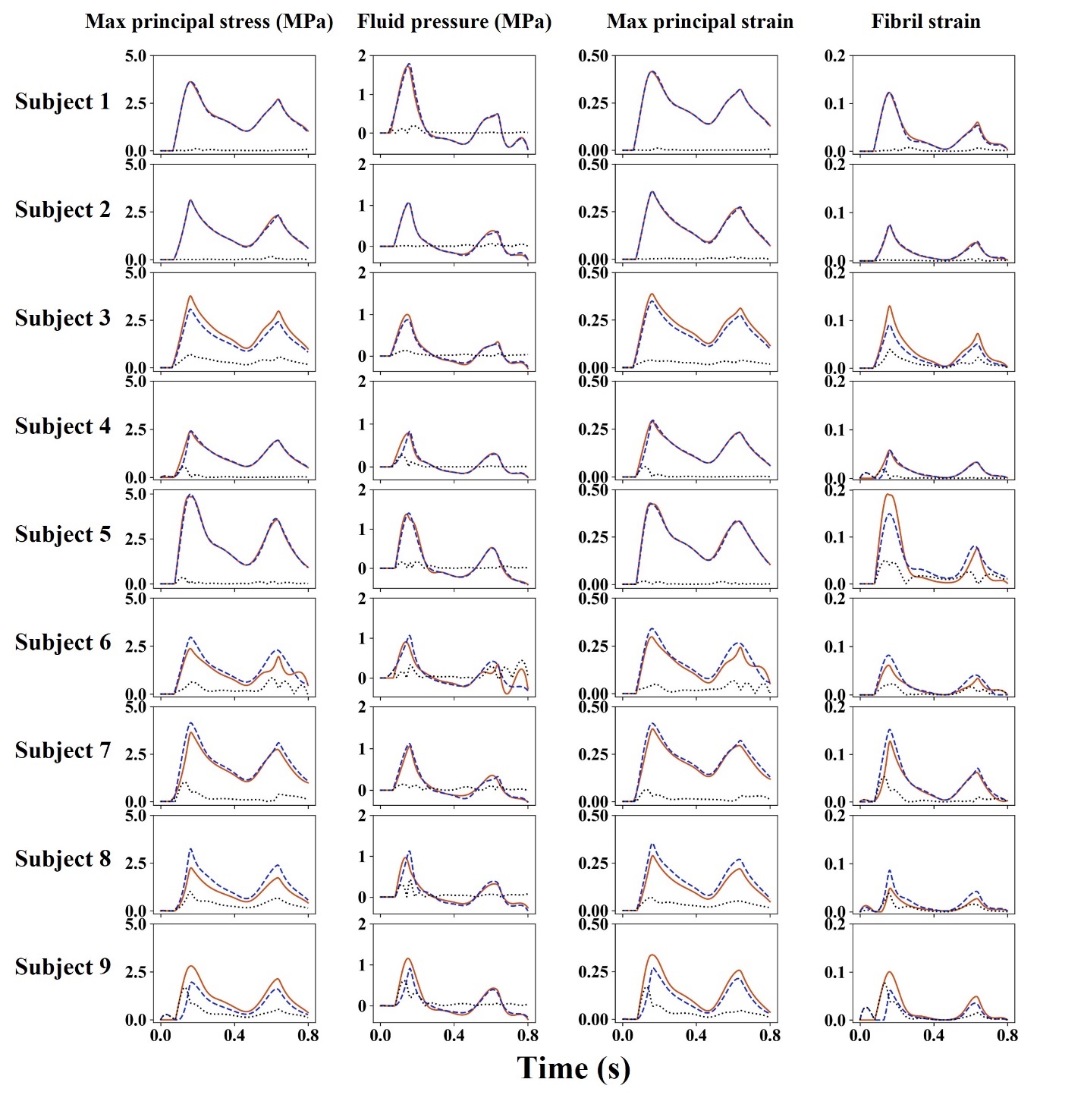


Figure 9S. The **peak** values of the mechanical parameters in the **superficial zone** for all cartilage models. The solid and dashed lines represent the manual and semi-automated models, respectively. The dotted line is the absolute difference between the two models. Values were calculated from the contact region.


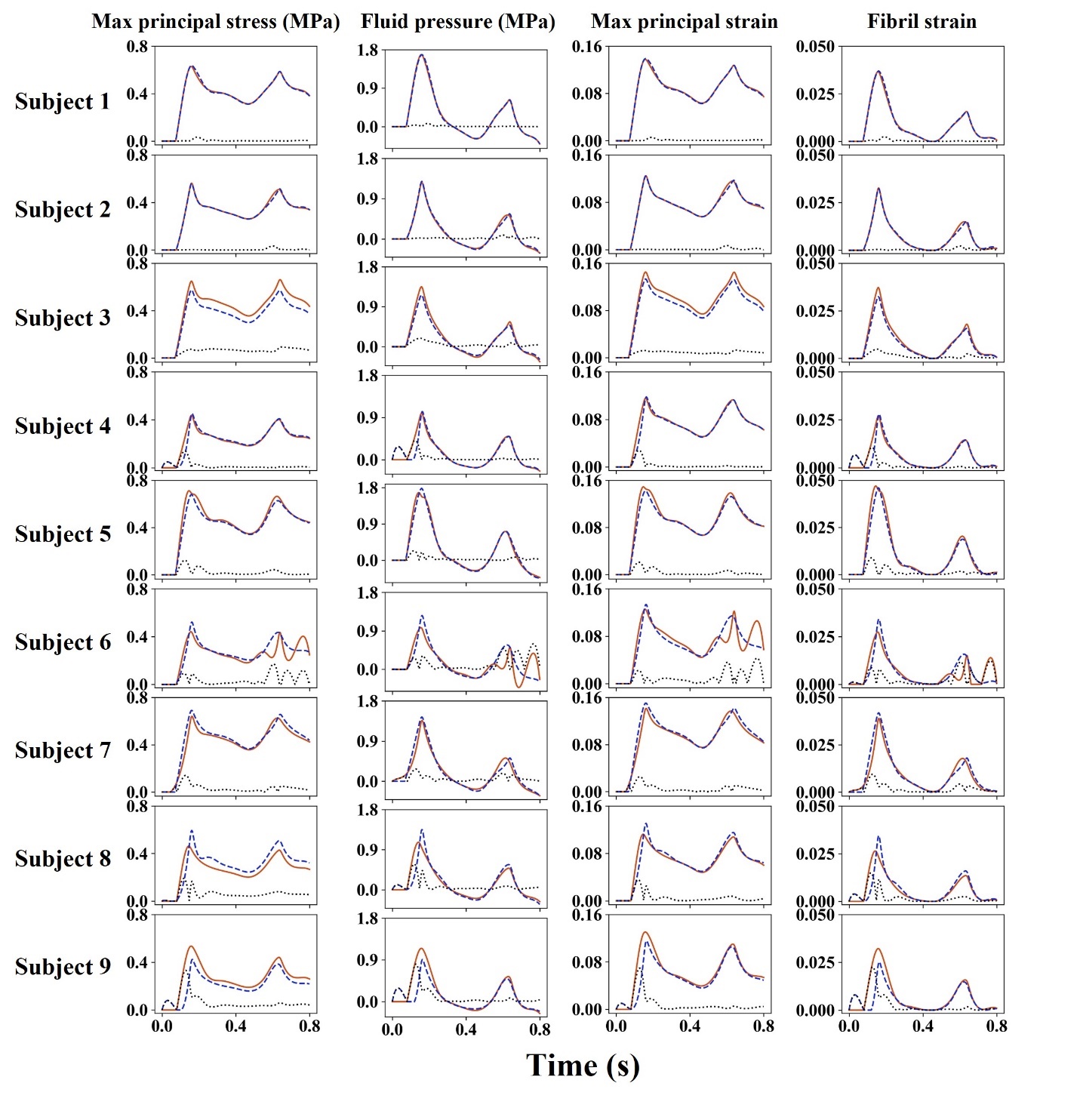


Figure 10S. The **average** values of the mechanical parameters in the **deep zone** for all cartilage models. The solid and dashed lines represent the manual and semi-automated models, respectively. The dotted line is the absolute difference between the two models. The contact region of the superficial zone was projected into the deep zone for calculating the parameters.


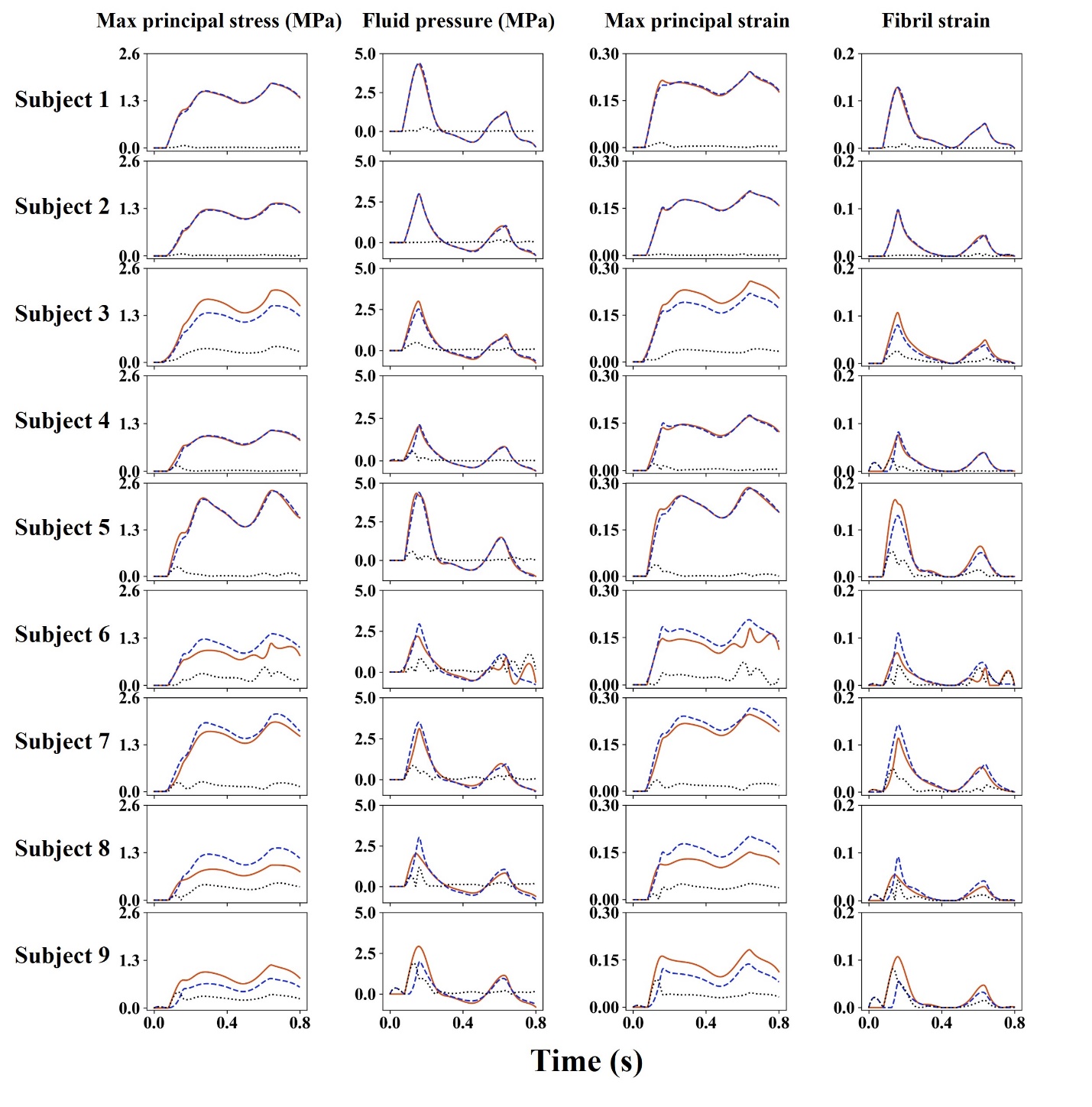


Figure 11S. The **peak** values of the mechanical parameters in the **deep zone** for all cartilage models. The solid and dashed lines represent the manual and semi-automated models, respectively. The dotted line is the absolute difference between the two models. The contact region of the superficial zone was projected into the deep zone for calculating the parameters.


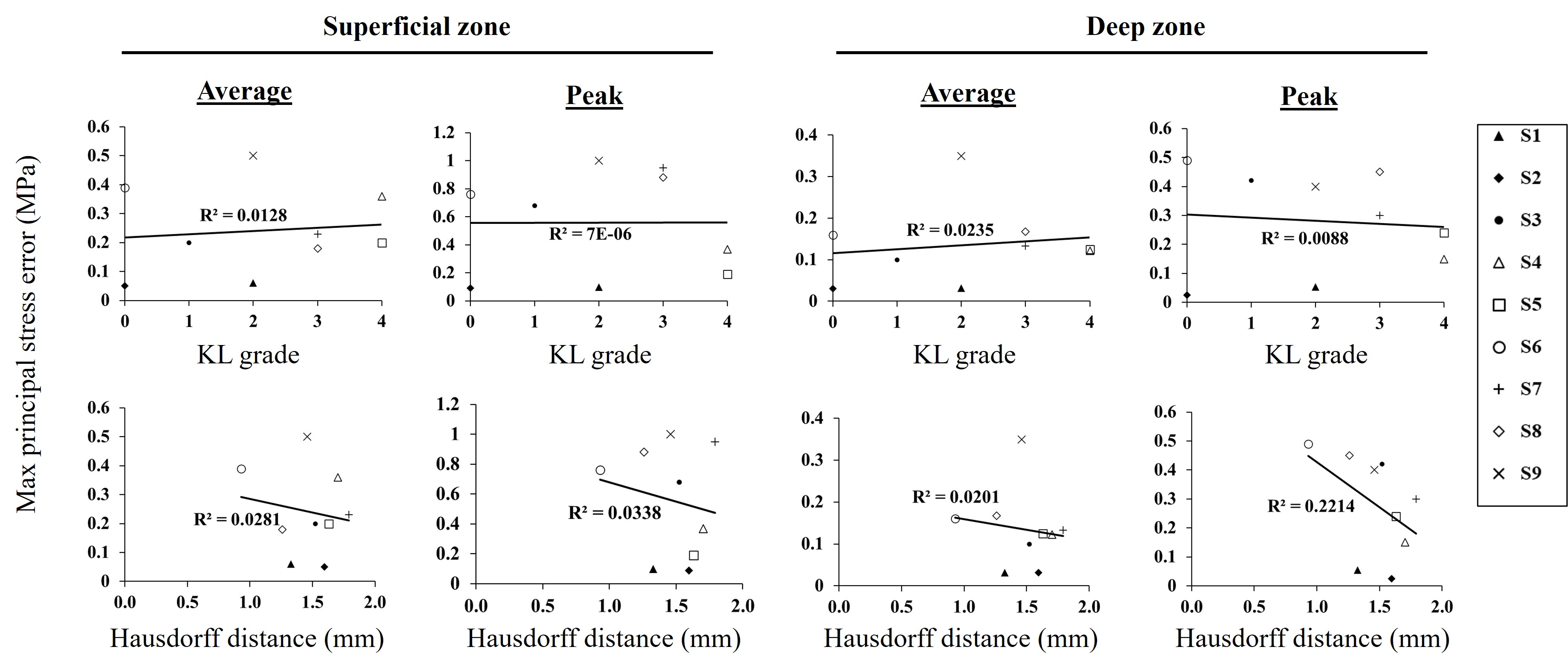


Figure 12S. Variation of maximum principal stress (MPa) against KL grade and Hausdorff distance (mm) for nine models. No significant correlation was observed between the error in predicting the mechanical responses by the semi-automated method and the KL grad and Hausdorff distance.
